# The Role of MicroRNAs in Lung Cancer Metabolism

**DOI:** 10.3390/cancers13071716

**Published:** 2021-04-05

**Authors:** Mohamed Iman Hidayat Nor Azizi, Iekhsan Othman, Rakesh Naidu

**Affiliations:** Jeffrey Cheah School of Medicine and Health Sciences, Monash University Malaysia, Jalan Lagoon Selatan, Bandar Sunway, Selangor Darul Ehsan 47500, Malaysia; mohamed.norazizi@monash.edu (M.I.H.N.A.); iekhsan.othman@monash.edu (I.O.)

**Keywords:** lung cancer, metabolic reprogramming, miRNAs, metabolism

## Abstract

**Simple Summary:**

Tumor cells exhibit dysregulation in metabolic activities compared with normal cells. Lung cancer is one of the most commonly diagnosed cancers globally. Studies have shown that miRNAs play important roles in metabolic reprogramming and our review aims to identify the roles of different miRNAs in metabolic aberrancies of lung cancer. Potentially, a few miRNAs could serve as biomarkers and therapeutic avenues depending on their significance to lung cancer metabolism.

**Abstract:**

MicroRNAs (miRNAs) are short-strand non-coding RNAs that are responsible for post-transcriptional regulation of many biological processes. Their differential expression is important in supporting tumorigenesis by causing dysregulation in normal biological functions including cell proliferation, apoptosis, metastasis and invasion and cellular metabolism. Cellular metabolic processes are a tightly regulated mechanism. However, cancer cells have adapted features to circumvent these regulations, recognizing metabolic reprogramming as an important hallmark of cancer. The miRNA expression profile may differ between localized lung cancers, advanced lung cancers and solid tumors, which lead to a varying extent of metabolic deregulation. Emerging evidence has shown the relationship between the differential expression of miRNAs with lung cancer metabolic reprogramming in perpetuating tumorigenesis. This review provides an insight into the role of different miRNAs in lung cancer metabolic reprogramming by targeting key enzymes, transporter proteins or regulatory components alongside metabolic signaling pathways. These discussions would allow a deeper understanding of the importance of miRNAs in tumor progression therefore providing new avenues for diagnostic, therapeutic and disease management applications.

## 1. Introduction

According to The International Agency for Research on Cancer (IARC), lung cancer (LC) is the most commonly diagnosed cancer type, enveloping 11.6% of total cancer cases [1]. Statistics show a gender bias leaning towards men in terms of incidence and mortality of LC cases. These LC cases show a wide geographical variation, respectively, with gender-dependent variance, which could be attributed to the country’s socioeconomic standing as risk factors such as occupational hazards, lifestyle, pollution, environmental stress and genetic predisposition play a critical role in LC tumorigenicity [2]. LC can be categorized into two major groups: small cell lung carcinoma (SCLC) and non-small cell lung carcinoma (NSCLC). The latter represents 85% of all LC cases and can be further subcategorized into large cell carcinoma, squamous cell carcinoma (SCC) and adenocarcinoma (ADC). The SCC tends to arise in the main bronchi meanwhile the ADC tends to occur in the peripheral bronchi and alveolar tissues. These subtypes can metastasize to extrathoracic organs in advanced cancer stages. Growing evidence suggests lung cancer to be histologically and molecularly heterogenous even within the same histological subtypes [3,4].

There are ten distinct features characteristic of cancer cells that depict tumorigenicity. One such prominent feature is cellular energetic dysregulation, an adaptive ability to circumvent conventional metabolism to support tumor pathogenesis [5]. Under normal conditions, glucose will be converted into pyruvate and fed into the tricarboxylic acid cycle then further reduced to carbon dioxide by the mitochondria in the presence of oxygen. However, in cancer cells, even in the presence of oxygen glucose is reduced through glycolysis and this aberrant process is termed aerobic glycolysis and was coined by Otto Warburg [6]. The event of aerobic glycolysis was initially challenged as it produces significantly less ATP; therefore, it may not support the high proliferative rate and energy demands of cancer cells. However, this was later found to support other branches of metabolic processes. By channeling glucose through aerobic glycolysis, although producing lesser ATP, this process generates higher levels of intermediaries supporting other branches of metabolism to meet rapid cellular proliferative demand alongside generating ATP through alternative mechanisms switching between oxidative phosphorylation and aerobic glycolysis [7]. Metabolic reprogramming is regulated through a complex network, namely, the increased expression of key metabolic enzymes, mutations on metabolism driver genes, the activation of signaling pathways, enhanced mitochondrial biogenesis, oxidation of fatty acids and the suppression of oxidative phosphorylation (OXPHOS). Growing evidence has shown that miRNAs play an important role in the metabolic deregulation of lung cancer cells. Enhanced lipid and glutamine metabolism also occurs in lung cancer metabolism reprogramming to meet the high energy and proliferative demands of cancer cells [8,9,10].

MiRNAs are short, non-coding RNAs usually about 18–25 nucleotides in length and have the ability to regulate gene expression either by mRNA degradation or by translation inhibition by binding to RNA-induced silencing complex (RISC). Post-translational gene modulation is elicited by the 3′-untranslated region (3′-UTR) sequence of the target messenger RNA (mRNA). MiRNA expression dysregulation involves the dysfunctional miRNA biogenesis machinery. These envelope the regulatory enzymes such as Drosha, DGCR8, Dicer and transport proteins. These components are critical in ensuring proper miRNA maturity [11]. Moreover, whole genome sequencing has shown that high amounts of miRNA genes are located in cancer-associated genomic regions that are responsible for tumor-suppressive genes, oncogenes and common breakpoint regions. Amplification, deletion and translocation of these genomic sites are responsible for the altered expression of miRNAs [12]. In addition, a high proportion of miRNA loci are associated with the CpG island, showing a DNA methylation-based epigenetic regulation on miRNA expression. Atypical tumor suppressor gene methylation, global DNA hypomethylation and alteration in histone modification patterns can result in differentially expressed miRNA in cancer cells [13,14]. A major number of genes encoding for miRNAs are present in the introns or long non-coding RNA genes having their respective promoter and enhancer regions. Gene transcription solely for miRNA expression are performed by RNA polymerase II and transcribed as a polycistronic message as miRNA genes present in a clustered form. Several miRNA genes can be governed by a vast number of RNA polymerase II-associated transcription factors by a single factor, usually via a complex feedback loop and feed-forward loop.

MiRNA regulates various functions including cell proliferation, metabolism, apoptosis and survival, which are commonly dysregulated mechanisms in cancer. In cancer cells, miRNA genes can either be amplified or deleted, which results in miRNA expression dysregulation. In advanced NSCLC, a common deletion was observed on *5q33*, resulting in the decreased expression of miR-143 and miR-145 [11,15,16]; meanwhile, amplifying the miR-17-92 cluster gene caused an increased expression of respective miRNA [15]. It was found that miRNA genes can be identified in a cancer-associated genomic region, which could carry tumor suppressor genes to which altercations from fragile sites and breakpoint genes would result in the loss of heterogeneity and support tumorigenesis. In this review, we focus on the role of miRNAs in lung cancer metabolism. To date, investigations into their roles, especially in metabolic signaling pathways, requires more attention.

**Figure 1:**
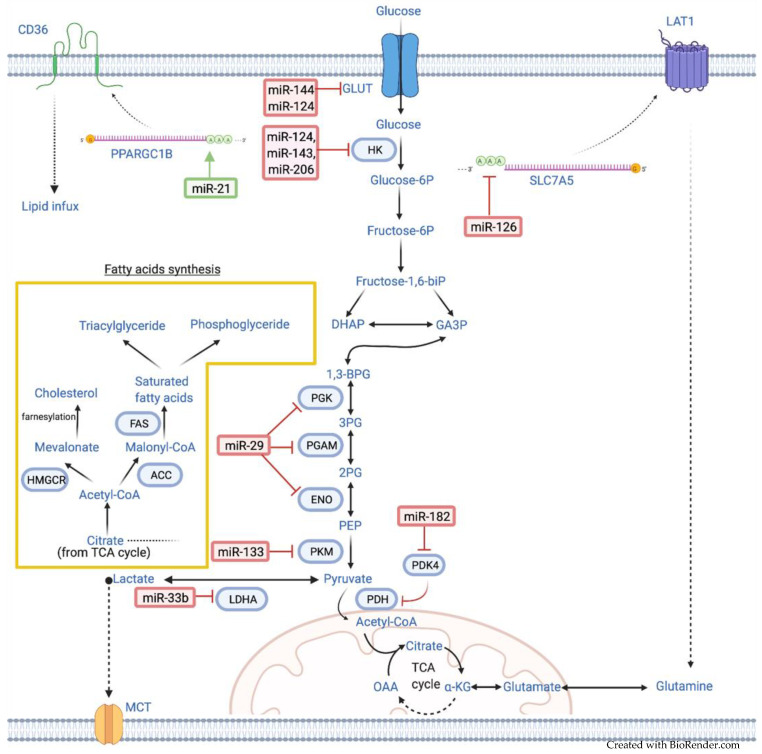
MicroRNAs regulate lung cancer metabolism by targeting key enzymes and protein mRNAs. The red sign indicates inhibition while the green arrow indicates promotion. DHAP: dihydroxyacetone phosphate, GA3P: glyceraldehyde-3-phosphate, BPG: 1,3-bisphosphoglycerate, 3PG: 3-phosphoglycerate, 2PG: 2-phosphoglycerate, PEP: phosphoenolpyruvate, α-KG: α-ketoglutarate, HK: hexokinase, PGK: phosphoglycerate kinase, PGAM: phosphoglyceromutase, ENO: enolase, PKM: pyruvate kinase, PDH: pyruvate dehydrogenase, PDK: pyruvate dehydrogenase lipoamide kinase, LDHA: lactate dehydrogenase, ACC: acetyl-CoA carboxylase, HMGCR: 3-hydroxy-3-methylglutaryl-CoA, FAS: fatty acid synthase.

Therefore, the mechanistic action of miRNAs in lung cancer warrants further research to understand the therapeutic and prognostic value better. The summary of miRNAs and their key target metabolic enzymes including the associated signaling pathways involved in lung cancer metabolism are shown in Table 1 and Figure 1 [16].

## 2. MiRNAs Involved in Lung Cancer Metabolism

Metabolic reprogramming is a key feature in tumorigenesis to ensure cell survival, proliferation and growth. This metabolic shift provides cancer cells with the necessary nutrients and intermediaries to support many biological processes such as rapid ATP generation and enhanced biosynthesis and allows a fast adaptation to the microenvironment. Research has shown that miRNAs are capable of either directly or indirectly affecting changes on metabolic enzymes, signaling pathways or modulating transcription factors [35].

### 2.1. MiRNAs Associated with Glucose Metabolism

The amplified cellular proliferative ability of cancer cells requires a high energy demand to generate sufficient nutrients, cellular components and microenvironment modifications to sustain tumorigenicity, which is supported by metabolic dysregulation [36]. As previously mentioned, the “Warburg effect” was first coined by Otto Warburg when they observed the exaggeration of glycolysis even in the presence of sufficient oxygen. Initially thought to compensate for high ATP demands, it led to significant discoveries. The escalated aerobic glycolysis also provides intermediaries to supply biosynthesis pathways such as protein, lipid and nucleotide synthesis alongside intermediaries to replenish the citric acid cycle such as glutamine [37], which provides an alternate source to TCA progression and ATP generation. The metabolic reprogramming of glycolysis in cancer involves alterations to the glucose transport protein (GLUT) or glycolytic enzymes such as hexokinase (HK), pyruvate kinase (PKM) and enolase (ENO) [9]. The glucose transporter mediates the transmembrane migration of glucose into the cell, which allows for a higher glucose intake by cancer cells. An increased expression or activity of glycolytic enzymes results in higher metabolic rates by driving forward the regeneration of metabolic intermediaries.

#### 2.1.1. MiR-144

Solute carriers are embedded proteins responsible for transporting nutrients, ions and metabolites across cells. Glucose can be carried by two solute carrier proteins via active transport by the SGLT transporter protein (encoded by SLC5A gene family) and facilitative transport via the GLUT transporter protein (encoded by SLC2A gene family) [38]. Additionally, the GLUT protein family consists of 14 proteins. The two most common types that are aberrantly found in lung cancer are GLUT1, GLUT3 or both and have been identified to have a high affinity with and turnover rate of glucose [39]. MiR-144 is commonly found to be downregulated in various cancer types; similarly in NSCLC, which has tumor-suppressive properties. It was recorded that miR-144 has a direct inverse correlation with the glucose transporter GLUT1 whereby it causes an increase in glucose uptake and lactate secretion in NSCLC, therefore accelerating cell proliferation in cancer cells by supporting the accelerated energy demands [17].

#### 2.1.2. MiR-124

Glycolytic enzymes regulate the glucose flux into the cells and, more importantly, the hexokinases (HKs), which are responsible for the first-step reaction into committing the glucose molecule to the metabolic activity by phosphorylating glucose into glucose-6-phosphate (G6P) [40]. HK1 and HK2 are known as high affinity enzymes and while distribution varies in different tissue, the dysregulated expression of HK2 is marked as a tumor indicator [41]. Studies have been done on the cancer cell lines and mice models to indicate that HK2 is exclusively upregulated in lung cancer [42]. MiR-124 is an example of a tumor-suppressing miRNA that inversely regulates the glycolytic rate, lactate production and ATP generation. A low expression of miR-124 in lung cancer cells shows a significant increment in glucose consumption and ATP production [18] by upregulating GLUT1 and HK2 expressions, both of which are key enzymes in the rate-limiting step of the glycolytic pathway. MiR-124 was also found to affect p-Akt1 and p-Akt2 subunits negatively; the claim is supported when the increased expression of Akt reverses the effect of miR-124 inhibition reflecting the involvement of the miR-124-Akt axis in the glycolysis regulation.

#### 2.1.3. MiR-143, MiR-206, MiR-1

In a study by Fang and colleagues, the relationship between HK2 enzyme, the first rate-limiting step in glycolysis, drives the Warburg effect and miR-143 was found to be inversely related [19]. MiR-143 was found to be downregulated in lung cancer. The upregulation of miR-143 causes the downregulation of HK2 enzymes in lung cancer cells subsequently causing a significant reduction in glucose consumption and lactate production while the shRNA mediated silencing of HK2 also shows a similar response of reduced colony formation and declining cell proliferation together with impeded glucose consumption. The effects of ShRNA mediated HK2 silencing and enhanced HK2 expression restoring glucose consumption, proliferative ability and colony formation after miR-143 mediated inhibition displays a close interaction between HK2 and miR-143. MiR-206 is also a tumor-suppressive miRNA by modulating the HK2 expression by binding on the 3′-UTR of HK2 [20]. The downregulation of miR-206 in NSCLC leads to a higher expression of HK2, an increased glucose uptake and lactate production and ATP generation to meet cell proliferative energy demand. Consequently, this effect will also cause an incline in the colony-forming capacity, cell proliferation and migration. Additionally, the expression of miR-206 and miR-1 in lung adenocarcinoma cells is significantly downregulated resulting in glucose metabolism reprogramming. These miRNAs are responsible for the regulation of several genes of key pentose phosphate pathway (PPP) enzymes such as glycerol-3-phosphate dehydrogenase (GPD2), glucose-6-phosphate dehydrogenase (G6PD), transketolase (TKT) and 6-phosphogluconate dehydrogenase (6PGD). The oxidative branch of PPP also generates NADPH, a reducing power for nucleic acids and fatty acid synthesis and maintaining redox balance. This regulatory mechanism is achieved by the NRF2/KEAP1 axis. KEAP1 is a regulatory component to the NRF2/KEAP1 complex. During oxidative stress, KEAP1 dissociates from NRF2 therefore stabilizing and activating NRF2. In turn, NRF2 inhibits the expression of miR-1 and miR-206 [43].

#### 2.1.4. MiR-133

Another example of a tumor-suppressive miRNA is miR-133, which was found to be downregulated in radiation-resistant A549 lung cancer cells relative to radiation-sensitive A549 cells [21]. Radio-sensitivity has been linked to a variable level of dysregulation of glucose consumption and lactate production in cancer cells. MiR-133 was found to target and is inversely correlated with the PKM2 expression profile in NSCLC. A study has shown that radio-sensitive and radio-resistant NSCLC that expresses miR-133 at a low level indicates a higher level of PKM2 expression level, enhanced glucose consumption and lactate production [44]. PKM2 is a rate-limiting enzyme that converts phosphoenolpyruvate and ADP into pyruvate and ATP, respectively, in the glycolytic pathway. Furthermore, PKM2 serves multiple functions such as a metabolic enzyme, protein kinase and gene co-activator, which regulates cell proliferation and apoptosis [45].

#### 2.1.5. MiR-29

A study done by Muniyappa and colleagues using two-dimensional difference gel-electrophoresis (2D DIGE) protein profiling demonstrated that miR-29 is downregulated in highly invasive and poorly differentiated lung carcinoma cells [22] and targets several metabolic enzymes involved in various pathways including enolase-alpha (ENO1), phosphoglycerate kinase 1 (PGK1), phosphoglycerate mutase (PGAM1), cytidine monophosphate kinase (CMPK1) and lysophospholipase II (LYPLA2). ENO1, PGK1 and PGAM1 are involved in different stages of glycolysis. Firstly, ENO1 is a metalloenzyme that catalyzes the conversion of 2-phosphoglyceric acid to phosphoenolpyruvic acid [46]. Furthermore, PGK1 catalyzes the conversion of 1,3-bisphosphoglycerate to 3-phosphoglycerate while generating ATP from ADP. Meanwhile, PGAM1 catalyzes the conversion of 3-phosphoglycerate to 2-phosphoglycerate. This step of glycolysis is downstream to many intermediates that supply anabolic metabolism [47].

### 2.2. MiRNAs Associated with Lipid Metabolism

Lipid metabolism dysregulation is essential in cancer progression. Fatty acid biosynthesis is supplied by either de novo lipogenesis or from dietary sources. They are vital for membrane synthesis, redox balance and cell survival [48,49]. Fatty acids (FAs) are crucial building blocks in actively proliferating cells, energy production and storage and even cellular signaling [50]. Cancer cells upregulate lipid metabolism, more commonly de novo lipogenesis, to support this oncogenic feature. Citrate from the tricarboxylic acid (TCA) cycle mainly provides the acetyl groups in fatty acid biogenesis. The coupling of acetyl and malonyl groups form fatty acid chains, which then undergo further processes to supply cell membrane building blocks [50]. However, lipid metabolism is also supported by glucose metabolism by feeding the glycolysis intermediary, glyceraldehyde-3-phosphate, into lipogenesis. In lung cancer, there are several alterations in the deploy of lipid metabolism cancer cells to propagate tumorigenicity. Most common is an increased de novo lipogenesis by potentiating the expression of lipogenic enzymes such as fatty acid synthase (FAS), stearoyl CoA desaturase 1 (SCD1) and ATP citrate lyase (ACLY). A higher FAS expression in lung cancer has been correlated to a poor prognosis and low survival [51] and is indicative of cancer aggressiveness [52]. Meanwhile SCD1 is a key enzyme in maintaining a proper ratio of saturated fatty acids (SFA) and monounsaturated fatty acids (MUFA) to ensure cell function and fluidity. The upregulation of SCD and SCD1 gene expression is a key factor for lung cancer-initiating cells and marked poor prognosis [53] along with increased metastasis in in vitro and in vivo studies [54]. ACLY is one of the enzymes linking glucose and lipid metabolism. In in vitro studies, a higher expression of ACLY demonstrated a poorer overall survival [55] while the inhibition of ACLY showed the induction of apoptosis and cell differentiation [56]. Moreover, apart from relying on lipogenesis, lung cancer cells also increase the uptake of extracellular fatty acids. Fatty acids transport protein such as FABP4, a cytoplasmic transport protein for fatty acids [57].

#### 2.2.1. MiR-33b

MiR-33b is significantly downregulated in NSCLC. Its overexpression has a marked decrease in glucose consumption, lactate metabolism and ATP production. This effect is due to the lactate dehydrogenase (LDHA) targeting ability of miR-33b in NSCLC, which is an essential enzyme in converting pyruvate to lactate [23]. MiR-33b downregulates LDHA expression therefore reducing lactate production. LDHA plays a significant role in glycolysis; in this case, promoting cancer progression via aerobic glycolysis. Enhanced lactate production generates NAD+, lowering the tumor microenvironment’s pH for invasion and aiding in immune evasion [58].

#### 2.2.2. MiR-21

Another miRNA regulating lipid metabolism is miR-21, which has been studied to promote cancer progression by targeting the tumor suppressor genes involved in apoptosis, proliferation and invasion [59] specifically in drug-resistant human lung adenocarcinomas through HBP1 [25]. The relationship between miR-21 and CD36, a lipid and fatty acid receptor, has been studied in multiple cancer types [60,61]. In human non-small lung carcinomas, the upregulation of miR-21 significantly enhanced proliferation and migration. Furthermore, the intracellular lipid and lipid metabolic enzymes increased approximately three-fold. Meanwhile, the effect of miR-21 was impeded with the suppression of CD36, which suggests miR-21 acts on lipid metabolism through CD36 with the involvement of PPARGC1B [62]. In normal cells, CD36 is a transmembrane glycoprotein expressed on the surface of multiple cell types where, depending on the tissue type, it serves different purposes. CD36, also known as fatty acid translocase (FAT), is responsible for maintaining lipid homeostasis, angiogenesis and metastasis in cancer cells [63]. Meanwhile, PPARGC1B is a master regulator of mitochondrial biogenesis and is a dynamic system mediated by a feedback loop to cater for the metabolic needs of cells [64].

#### 2.2.3. MiR-182

MiR-182 was found to be overexpressed in A549 cells and supports lung tumorigenesis with pyruvate dehydrogenase lipoamide kinase isozyme (PDK4) being its direct target. The upregulation of the miR-182 expression in A549 cells promotes cell proliferation and colony-forming abilities and drives cell cycle progression by causing cells to exit the G_0_/G_1_ phase. The inhibitory effect of miR-182 on PDK4 leads to the upregulation of pyruvate dehydrogenase (PDH), which encourages de novo lipogenesis and increases triglycerides levels. Subsequently, the ectopic level of lipogenesis increases the ROS level. ROS oxidates and inhibits c-Jun N-terminal kinase (JNK) inactivating phosphates [65]; this activates JNK signaling; which implicates the miR-182/PD4 axis dysregulation and elicits its effect by enhancing de novo lipogenesis through PDH upregulation and ultimately the activation of the JNK signaling pathway [26]. PDH acts at the checkpoint between glycolysis and other metabolic pathways, either branching off into amino acid metabolism, increasing lipogenesis or channeling substrates into the TCA cycle. This enzyme catalyzes the irreversible oxidative decarboxylation of pyruvate into Acetyl-CoA, then feeds into the TCA for energy production [66]. Meanwhile, PDK4 responds to the metabolic demands of tissues and is a regulator of PDH activity; this PDK/PDH axis is commonly dysregulated in cancer [67].

### 2.3. MiRNAs Associated with Amino Acid Metabolism

The importance of amino acid metabolism has been highlighted in cancer progression especially the involvement of glutamine. The TCA cycle is imperative in supplying citrate as an Acetyl-CoA precursor for the fatty acid synthesis and replenishing oxaloacetate levels in the cycle. However, high demands from other metabolic pathways during tumorigenesis may diminish the citrate pool. The withdrawal of TCA intermediaries causes an influx of carbon to compensate for the increase of efflux [16]. Amino acid metabolism is commonly upregulated to meet cellular demands especially in highly proliferating cells for protein synthesis. One of the most important aspects of amino acid metabolism is glutamine metabolism [68]. Glutamine is an abundantly available amino acid that contributes to an array of pathways including energy production, macromolecule synthesis and cell signaling. Glutamine is transported into the cytoplasm via its respective transporter proteins then converted to glutamate by glutaminase [69]. Glutamate can be channeled into the mitochondria to generate α-ketoglutarate, which supports the TCA cycle in energy generation or undergoes transamination in the cytoplasm to synthesize non-essential amino acids. Furthermore, glutamate is important for replenishing fumarate, malate and citrate. The influx of α-ketoglutarate could increase the citrate level by a forward reaction in the TCA cycle but glutamine-derived citrate could also be generated by the reverse reaction by the action of isocitrate dehydrogenase-1 (IDH-1) and aconitase 1 (ACO1) [70].

#### MiR-126

MiR-126 was found to inhibit cell proliferation by halting progression in the G_1_ phase and has an inverse relationship with SLC7A5, one of the target proteins that regulates the proliferative effect of SCLC cells [27]. SLC7A5 is a light-chain protein in a sodium-independent transporter allowing an amino acid exchange and, more importantly, the glutamine-leucine exchange [71]. Essentially, this supplies amino acids to cancer cells for accelerated proliferation while maintaining intracellular leucine levels, which, in turn, activate mTOR [72]. It is important to note that miR-126 can also act on the PI3K pathway by targeting the p85β subunit via a translation inhibition [73].

### 2.4. MiRNAs Involved in the Metabolic Signaling Pathway

A study investigated the target gene of miR-144-3p in correlation with its downregulation in lung cancer. Using a Gene Expression Profiling Interactive Analysis (GEPIA), overlapping highly expressed genes in a lung adenocarcinoma and a lung squamous cell carcinoma were identified as targets of miR-144-3p. Inclusive of all three aspects, miR-144-3p was found to prominently target genes involved in nucleobase-containing small molecule metabolic processes, mitochondria envelopes and transferase activity alongside other target genes involved in processes such as carbohydrate metabolism, glycoprotein metabolism, protein localization into the nucleus, the regulation of nucleobase-containing small molecule metabolic processes and kinase binding [17]. The differential expression of miRNAs has been linked to an intervention in metabolic signaling pathways to support cancer pathogenesis and progressions.

#### 2.4.1. PI3k/Akt/PTEN Pathway

The recruitment of class I phosphoinositide-3-kinase (PI3K) is enabled by the activation of receptor tyrosine kinase (RTK) or G-protein-coupled receptors. This leads to the phosphorylation of phosphatidylinositol-(4,5)-bisphosphate (PIP_2_) into phosphatidylinositol-(3,4,5)-triphosphate (PIP_3_). The amassing of PIP_3_ allows the localization of Akt to the plasma membrane and subsequent activation by mTORC2 or PDK1 [74]. Akt is a crucial downstream effector of PI3K that regulates an array of functions such as cell survival, proliferation and metabolic processes [75]. The phosphatase and tensin homolog (PTEN) is a negative regulator of PI3K by inhibiting the phosphorylation of PIP_2_ to PIP_3_. Tumorigenic adaptations involve aberrantly regulating PTEN expression, PIK3CA genes (a PI3K regulatory subunit) and Akt phosphorylation. This pathway mediates glycolytic signals including glucose transporter proteins, glycolytic enzymes and lipogenesis [76]. The central activator in this pathway is Akt, which can affect multiple downstream effectors to promote glucose, nucleotides and lipid metabolism and restore redox balance. For example, Akt can inhibit the Forkhead box O (FOXO) family [77] while activating HIF [78] and stabilizing the c-Myc protein [79] to promote glucose metabolism. Meanwhile, Myc and ATF4 [80] activation would potentiate nucleotide biosynthesis. Moreover, Akt also activates the SREBP family protein to support lipid synthesis [81] and the pentose phosphate pathway. The acceleration of lipid synthesis and Akt mediated NRF2 activation restores the redox balance by supplying reducing agents for biosynthetic processes.

The negative regulation of PTEN and by extension Akt activation was observed in several miRNAs in lung cancer to modulate metabolism. These effects lead to a higher glucose uptake and lactate production and an increased proliferation and migration of the metastatic and invasive capacity of cancer cells. Examples of these miRNAs are miR-21, miR-155 [24] and miR-26 [82], which target and inhibit PTEN subsequently allowing the continued activation of Akt. In NSCLC, the overexpression of miR-21 significantly inhibits FBP-1 expression leading to an increase in glucose consumption and lactate production while suppressing oxidative phosphorylation (OXPHOS) [83]. Meanwhile, the overexpression of miR-155 targets HK2 and promotes glycolysis in NSCLC [84].In addition, miR-125b was upregulated in lung cancer cells correlating with a higher expression of p-Akt. Interestingly, miR-125b was also found to increase the protein expression of GSK-3β, a serine-threonine protein kinase that modulates multiple downstream pathways, which include metabolic and signaling proteins that increases glycogen and protein synthesis [85]. This leads to an increased cell proliferation while inhibiting apoptosis via the Wnt/β-catenin pathway [28]. This relationship was also observed between miR-21, PTEN and Wnt/β-catenin [86]. Additionally, miR-181a-5p shows an anti-cancer ability by regulating aerobic glycolysis and lipid contents by binding to the 3′-UTR of SIRT1 and ACLS4 [87].

#### 2.4.2. c-Myc Pathway

Under normal circumstances, c-Myc is stringently regulated and expressed in low levels except in the presence of developmental or mitogenic positive signals. However, c-Myc is commonly found to be dysregulated in cancer cells via gene mutation, mRNA translation disruption or alteration to c-Myc protein stability [88]. The expression level of c-Myc may oscillate between oncogenic and tumor-suppressive as it regulates cell transformation and metabolism while being able to induce apoptosis at a certain expression level, warranting the term ‘master regulator’ due to its dichotomy in providing a promising therapeutic foundation [89]. Downstream effects of c-Myc include ribosome and mitochondrial biogenesis, cell cycle progression, protein translation and metabolism. Aberrancy in c-Myc regulation serves as a major metabolic reprogramming in cancer, affecting glucose, glutamine and serine metabolism [90].

A study has shown that in docetaxel-resistant lung adenocarcinoma cells, the expression level of miR-451 is significantly downregulated leading to enhanced migration and invasion phenotypes. A further gene profile investigation dictates that c-Myc is the direct target to miR-451 and their expression profiles are inversely correlated. The loss of miR-451 or overexpression of c-Myc leads to GSK-3β inactivation, which subsequently amplifies the mesenchymal-epithelial transition (MET) phenotype [29].

#### 2.4.3. P53 Pathway

Tumor suppressor p53 is a key regulator to diverse biological functions such as controlling cell cycles, apoptosis and, more importantly, cellular metabolism. In normal cells, p53 levels are kept low but increase during stress. P53 is a commonly mutated gene in cancer and tightly regulated by ubiquitination and protein degradation. Mutations on p53 are more commonly missense mutations leading to loss-of-function or gain-of-function, acquiring oncogenic properties that are independent of the wild-type. While the p53 expression level directly mediates biological functions, it also acts as a transcription factor affecting the downstream effector to promote tumorigenesis [91]. P53 plays an important role in regulating metabolism. For example, p53 inhibits glycolysis through multiple ways, i.e., downregulating the expression of glucose transport proteins, the GLUT family, on the transcriptional level [92]. P53 also downregulates the expression and protein levels of glycolytic enzymes such as pyruvate dehydrogenase kinase 2 (PDK2) and phosphoglycerate mutase (PGM) [93,94] while also inducing glycolysis negative regulators such as Parkin (PARK2) [95]. Meanwhile, in lipid metabolism p53 inhibits fatty acid synthase (FASN) and ACLY [91]. These features are achieved by p53 direct regulation on metabolic enzymes or intermediate activators such as mTORC1, SREBPs and NF-κB [96]. P53 levels in normal cells are kept low but increase when stress is introduced.

P53 is another regulatory pathway for miRNA expression that is encoded by the tumor suppressor TP53 gene, which is commonly mutated in cancer cells. Much like p53, miR-34 is related to the regulation of cell cycle progression, senescence and apoptosis; together, they form a complex signaling network [31]. P53 has been found to promote the expression of miR-34 to induce apoptosis via binding to the miR-34a gene promoter site [30]. MiR-34, in turn, promotes the expression of p53 by SIRT1 targeting, which is a p53 negative regulator [97].

## 3. Relationship between MiRNAs and Hypoxia in Lung Cancer

Hypoxia refers to oxygen supply deprivation, commonly found in most tumors and prominently in solid tumors where the oxygen demand to support rapid cell proliferation, survival and metabolic activity surpasses available oxygen supply [98]. Cancer cells deploy an adaptive mechanism to alter their microenvironment to meet demands. Hypoxia is associated with poor prognosis and radiation and chemoresistance [99]. Therefore, targeting hypoxia provides promising therapeutic grounds. The hypoxia-inducible factor (HIF) is a critical transcription factor activated as an oxygen stress response. The HIF pathway further regulates an array of downstream effectors, i.e., gene expression and signaling pathway regulation ranging from survival and potentiating oncogenic features to cellular metabolism [100]. While the HIF expression, in part, is controlled by the expression profile of miRNAs, it is important to note that the HIF can also affect miRNA expression as a downstream effector.

### 3.1. MiR-210

The effect of miR-210 has been studied in high grade lung cancer cells and found to affect mitochondria via structural deregulation. This is achieved by the miR-210 mediated inhibition of complex I and complex II subunits NDUFA4 and succinate dehydrogenase (SDHD), respectively, which cause phenotypic disfigurement of the mitochondria and cristae arrangement. Although NDUFA4 inhibition does not reduce electron transport chain (ETC) complex activity, SDHD inhibition elicits reduced expression and activity along with reduced cell viability and increased apoptotic capacity via the activation of caspases [33]. Previous studies have shown miR-210 involvement in ISCU1/2, which is responsible for integrating iron-sulfur clusters into the enzymes involved in energy production via the TCA cycle and contributing to ETC activity [101]. A greater highlight of SDHD inhibition by miR-210 is the stabilization of HIF-1α, which induces angiogenesis. Increased HIF-1α stability explains the increased expression in high grade lung cancer cells that supports invasion and growth and plays a role in the increased glycolytic ability in cancer cells [102].

### 3.2. MiR-31-5p

Another miRNA that is commonly found to be overexpressed in cancer cells is miR-31-5p, which includes lung adenocarcinomas and non-small cell lung carcinomas. The aberrant expression of miR-31-5p was confirmed to support tumor progression and the expression level progressively increases as the cancer develops into later stages [34]. This miRNA promotes aerobic glycolysis via the HIF/FIH axis to amplify HIF transactivation. Factor-inhibiting HIF-1α (FIH) inhibits the transactivation of HIF [103] by preventing binding to the co-activator via hydroxylation of the HIF-1α subunit [104]. It is a direct target of miR-31-5p and their expression levels are inversely correlated. In lung cancer, the upregulation of miR-31-5p causes a significantly decreased FIH expression level that increases HIF activation thus driving the Warburg effect marked by increased pyruvate and lactic acid production. Ultimately, this leads to elevated cell proliferation and tumor growth [105]. This regulatory pathway can be observed in in vitro and in vivo studies using the A549 cell xenograft mouse model.

### 3.3. MiR-199a-5p, MiR-34

A study has shown that miR-199a-5p has a negative correlation with HIF-1α expression and Akt activation while miR-34 exhibits an inverse relationship with pAkt. A further study connected the different signaling components that stated that p53 is an upstream regulator of miR-34 while miR-199a-5p is downstream of Akt and miR-34 but upstream of HIF-1α [32]. The involvement of Akt activation and HIF-1α is a crucial element in lung cancer metabolic dysregulation given that Akt is upstream to many metabolism-regulating pathways and HIF-1α drives the Warburg effect forward. P53 also plays an important role in this model given that the majority of cancer types, including lung cancer, exhibit a mutated form of p53 [106].

### 3.4. MiR-21, MiR-200c, MiR-519c

MiR-21 has been shown to play a major role in oncogenesis by regulating aerobic glycolysis-related enzymes. In a study done by Jiang and colleagues, miR-21 was found to enhance the HIF1α expression level, which mediates radio-resistance in A549 cells and promotes the mRNA transcription of aerobic glycolysis-related enzymes, i.e., HK2, PKM2 and LDHA. This provides radio-protection to lung cancer cells and amplifies the Warburg effect [107]. MiR-200c was found to inversely regulate the HIF1α expression level on protein and the mRNA level in lung cancer. Subsequently, HIF-1α affects the gene transcriptional activity of glycolytic enzymes such as LDHA, PGK1, PKM, fructose-bisphosphate aldolase (ALDOA) and the hypoxia indicator carbonic anhydrase 9 (CA9). In A549 cells, HIF-1α was shown to promote cell migration in hypoxia and normoxia while inhibition by miR-200c negatively affected this ability [108]. MiR-519c has been proven to negatively regulate endogenous HIF-1α at post-translational level via the hepatocyte growth factor (HGF)/c-Met pathway in a hypoxic-independent manner. This axis affects the shift in angiogenic and metastatic ability in vitro and in vivo. It is also important to note that HGF regulates mature miR-519c contrary to the pre-miRNA and primary miRNA (pri-miRNA) form [109].

## 4. Future Perspective of MiRNA Targeted Lung Cancer Therapy and Clinical Implication of Existing Chemotherapies on MiRNA Expression

The advantageous properties of miRNAs such as multiple effector targets and short sequences allow for a broad oncogenic silencing and a low probability of miRNA mutations and make miRNAs an effective target therapy. Several studies have also shown that relatively small alterations in miRNAs could revert malignant phenotypes [110,111,112]. Deploying tumor-suppressive miRNA mimicking or inhibiting oncogenic miRNAs can be effective miRNA targeted therapies [113]. Several miRNAs have been studied for their therapeutic application in in vivo NSCLC such as miR-15/16 [114], miR-29b [115], miR-7 [116], miR-34a [117], miR-200 [118], miR-145 [119] and let-7 [120] using different delivery systems. While promising preclinical studies have shown the potential of miRNA mimics and anti-miRNA to restore the normal gene network, concerns such as delivery method, stability, safety profile and interaction with other treatment forms remain a hurdle for strategy implementation.

Distinct miRNA profiles have been studied to aid in diagnostic processes by allowing a classification between lung cancer subtypes and even between primary and metastasized tumors. Even at an early stage, lung cancer shows molecular heterogenicity that contributes to its high incidence rate. By studying the miRNA profile, it provides a better prediction for more effective therapies. In the context of tumorigenesis, miRNAs can be categorized into tumor-suppressive miRNAs and tumor-promoting miRNAs. Currently, two main strategies of equipping miRNAs as therapeutic targets are to either restore tumor-suppressive miRNA functions or impede tumor-supporting miRNAs [121]. There are several methods for restoring tumor-suppressive miRNAs including a vector-based delivery and global miRNA expression regulation using pharmaceutical agents. However, each method presents their own concerns. With a vector-based delivery, stability, permeability and off-target toxicity are factors that require attention [122,123]. With global miRNA expression regulation using clinical drugs, there is an issue with target specificity [124]. The most promising effort is using miRNA mimics as they reduce off-target toxicity and can be personalized. The use of an anti-sense oligonucleotide is the primary strategy for inhibiting tumor-promoting miRNAs. This approach includes antagomirs, locked nucleic acid (LNA) miRNAs and miRNA sponges [121]. Antagomirs and miRNA sponges face the same challenge in that they require a large dose to elicit significant effects [125]. On the other hand, LNA miRNAs have a high target affinity and can reduce off-target toxicity [126]. An example of this is Miravirsen, a LNA miRNA that targets miR-122 to treat Hepatitis C but the therapeutic success of this treatment shows potential for the treatment of other diseases including lung-associated diseases [127,128].

A few existing clinical drugs, chemical or synthetic, can potentially target miRNAs and alter their expression profile. For example, Gefitinib downregulates the expression of miR-155 in NSCLC [129] while Lapatinib suppresses lactate dehydrogenase activity via miR-133b in cisplatin-treated cells [130]. In A549 cells, treatment with cisplatin (CDDP) downregulates the expression level of miR-21 while elevating the level of MSH2, a DNA mismatch repair protein, which has a tumor-suppressive effect [131]. Meanwhile, curcumin inhibits the expression of miR-21 leading to the upregulation of PTEN [132]. Solasodine inhibits the invasive ability of A549 cells possibly by reducing the matrix metalloproteinase (MMP) and blocking the PI3K/Akt pathway and downregulates miR-21 [133]. CDDP also significantly upregulates HER2 through miR-125a and miR-125b in SBC-3 and SBC-5 cell lines [134]. It was observed that in A549 cells, apoptosis was induced in CDDP-treated cells by the regulating of miR-98 through the TP53 pathway, which led to a further increase in bcl-2 and decreased levels of miR-34 [135]. MiR-34a-5p is upregulated in CDDP-treated A549 and H460 cells, inducing apoptosis [136]. Furthermore, H1299 and H460 cells treated with Epigallocatechin gallate (ECGC) induces the expression of miR-210, therefore inhibiting cell proliferation via HIF-1α [137].

## 5. Conclusions

Lung cancer is one of the most commonly diagnosed cancers with a high incidence, morbidity and mortality rate. Efforts to advance our understanding of the underlying molecular mechanisms of lung cancer for better therapeutic opportunity, diagnosis and management have been relentless. This review highlights the crucial role miRNAs play in regulating the cellular metabolism of lung cancer. Metabolic reprogramming is a hallmark of cancer that is achieved through alterations in several major aspects of metabolism including the Warburg effect, lipid metabolism, glutamine metabolism and hypoxia. MiRNAs act as regulators by promoting or inhibiting effector components in response to stress. The interactions between miRNAs and signaling pathways are equally important as they contribute to tumorigenesis. These regulations by miRNAs confer other oncogenic phenotypes for cancer cell propagation such as survival, metastases and treatment resistance. The wide involvement of miRNAs in lung cancer cell pathology provides a promising avenue for biomarkers and target-specific treatment using miRNA mimics or inhibitors on metabolism-regulating genes and proteins to improve overall survival. Nonetheless, there is still a knowledge gap in lung cancer metabolism that is yet to be answered.

## Figures and Tables

**Table 1:** The list of miRNAs associated with lung cancer (LC) metabolic reprogramming.

Metabolic/Signaling Pathway	MiRNA	Up/Downregulated	Target	Function(s)	Reference
Glucose metabolism	miR-144	Downregulated	GLUT1	Enhances Warburg effect	[17]
	miR-124	Downregulated	GLUT1, HK2, p-Akt1/2	Enhances Warburg effect and ATP generation	[18]
	miR-143	Downregulated	HK2	Enhances Warburg effect	[19]
	miR-206	Downregulated	HK2	Enhances Warburg effect	[20]
	miR-133	Downregulated	PKM2	Enhances Warburg effect and radiation resistance	[21]
	miR-29	Downregulated	ENO1, PGAM1, PGK1, CMPK1	Facilitates glycolysis	[22]
Lipid metabolism	miR-33b	Downregulated	LDHA	Enhances aerobic glycolysis and tumor invasion	[23]
	miR-21	Upregulated	HBP1, CD36	Promotes proliferation and increase intracellular lipid influx	[24,25]
	miR-182	Upregulated	PDK4/PDH axis	Enhances de novo lipogenesis and JNK activation	[26]
Amino acid metabolism	miR-126	Downregulated	SLC7A5	Facilitates amino acid exchange and activate mTOR	[27]
PI3K/Akt/PTEN	miR-21, miR-155	Upregulated	SOCS1, SOCS6, PTEN	PTEN inhibition	[24]
	miR-125	Upregulated	p-Akt, GSK3β	PI3K/Akt and GSK3β/Wnt/β-catenin activation	[28]
c-Myc	miR-451	Downregulated	c-Myc, GSK3β	Enhanced metabolic dysregulation and migration	[29]
Hypoxia	miR-34	Upregulation	SIRT1	Facilitate mutant p53 expression via a feedback loop	[30,31]
	miR-199-5p			Activates HIF-1^α^	[32]
	miR-210	Upregulated	NDUFA4, SDHD	Mitochondria disfiguration and cristae formation	[33]
	miR-31-5p	Upregulated	FIH	Promotes hypoxia and enhances aerobic glycolysis	[34]

JNK: c-Jun N-terminal kinase, PTEN: phosphatase and tensin homolog, PI3K: phosphoinositide-3-kinase, HIF-1α: hypoxia-inducible factor 1-alpha, Wnt/β-catenin: Wnt/β-catenin, GSK3β: glycogen synthase kinase 3 beta, Akt: protein kinase B, p-AKT: phosphorylate protein kinase B.

## References

[1] Bray F., Ferlay J., Soerjomataram I., Siegel R.L., Torre L.A., Jemal A. (2018). Global cancer statistics 2018: GLOBOCAN estimates of incidence and mortality worldwide for 36 cancers in 185 countries. CA Cancer J. Clin..

[2] De Groot P.M., Wu C.C., Carter B.W., Munden R.F. (2018). The epidemiology of lung cancer. Transl. Lung Cancer Res..

[3] Onaitis M.W., Hanna J.M. (2013). Cell of origin of lung cancer. J. Carcinog..

[4] Lemjabbar-Alaoui H., Hassan O.U., Yang Y.-W., Buchanan P. (2015). Lung cancer: Biology and treatment options. Biochim. Biophys. Acta (BBA) Bioenerg..

[5] Hanahan D., Weinberg R.A. (2011). Hallmarks of Cancer: The Next Generation. Cell.

[6] Warburg O. (1956). On the Origin of Cancer Cells. Science.

[7] Ahn C.S., Metallo C.M. (2015). Mitochondria as biosynthetic factories for cancer proliferation. Cancer Metab..

[8] Chang L., Fang S., Gu W. (2020). The Molecular Mechanism of Metabolic Remodeling in Lung Cancer. J. Cancer.

[9] Li Z., Zhang H. (2016). Reprogramming of glucose, fatty acid and amino acid metabolism for cancer progression. Cell. Mol. Life Sci..

[10] Mendes C., Serpa J. (2019). Metabolic Remodelling: An Accomplice for New Therapeutic Strategies to Fight Lung Cancer. Antioxidants.

[11] Peng Y., Croce C.M. (2016). The role of MicroRNAs in human cancer. Signal Transduct. Target. Ther..

[12] Tagawa H., Seto M. (2005). A microRNA cluster as a target of genomic amplification in malignant lymphoma. Leukemia.

[13] Lopez-Serra P., Esteller M. (2011). DNA methylation-associated silencing of tumor-suppressor microRNAs in cancer. Oncogene.

[14] Weber B., Stresemann C., Brueckner B., Lyko F. (2007). Methylation of Human MicroRNA Genes in Normal and Neoplastic Cells. Cell Cycle.

[15] Hayashita Y., Osada H., Tatematsu Y., Yamada H., Yanagisawa K., Tomida S., Yatabe Y., Kawahara K., Sekido Y., Takahashi T. (2005). A Polycistronic MicroRNA Cluster, miR-17-92, Is Overexpressed in Human Lung Cancers and Enhances Cell Proliferation. Cancer Res..

[16] DeBerardinis R.J., Mancuso A., Daikhin E., Nissim I., Yudkoff M., Wehrli S., Thompson C.B. (2007). Beyond aerobic glycolysis: Transformed cells can engage in glutamine metabolism that exceeds the requirement for protein and nucleotide synthesis. Proc. Natl. Acad. Sci. USA.

[17] Chen Y.-J., Guo Y.-N., Shi K., Huang H.-M., Huang S.-P., Xu W.-Q., Li Z.-Y., Wei K.-L., Gan T.-Q., Chen G. (2019). Down-regulation of microRNA-144-3p and its clinical value in non-small cell lung cancer: A comprehensive analysis based on microarray, miRNA-sequencing, and quantitative real-time PCR data. Respir. Res..

[18] Zhao X., Lu C., Chu W., Zhang B., Zhen Q., Wang R., Zhang Y., Li Z., Lv B., Li H. (2017). MicroRNA-124 suppresses proliferation and glycolysis in non–small cell lung cancer cells by targeting AKT–GLUT1/HKII. Tumor Biol..

[19] Fang R., Xiao T., Fang Z., Sun Y., Li F., Gao Y., Feng Y., Li L., Wang Y., Liu X. (2012). MicroRNA-143 (miR-143) Regulates Cancer Glycolysis via Targeting Hexokinase 2 Gene. J. Biol. Chem..

[20] Jia K.-G., Feng G., Tong Y.-S., Tao G.-Z., Xu L. (2019). miR-206 regulates non-small-cell lung cancer cell aerobic glycolysis by targeting hexokinase 2. J. Biochem..

[21] Liu G., Li Y., Gao X. (2016). Overexpression of microRNA-133b sensitizes non-small cell lung cancer cells to irradiation through the inhibition of glycolysis. Oncol. Lett..

[22] Muniyappa M., Dowling P., Henry M., Meleady P., Doolan P., Gammell P., Clynes M., Barron N. (2009). MiRNA-29a regulates the expression of numerous proteins and reduces the invasiveness and proliferation of human carcinoma cell lines. Eur. J. Cancer.

[23] Zhai S., Zhao L., Lin T., Wang W. (2019). Downregulation of miR-33b promotes non-small cell lung cancer cell growth through reprogramming glucose metabolism miR-33b regulates non-small cell lung cancer cell growth. J. Cell. Biochem..

[24] Xue X., Liu Y., Wang Y., Meng M., Wang K., Zang X., Zhao S., Sun X., Cui L., Pan L. (2016). MiR-21 and MiR-155 promote non-small cell lung cancer progression by downregulating SOCS1, SOCS6, and PTEN. Oncotarget.

[25] Su C., Cheng X., Li Y., Han Y., Song X., Yu D., Cao X., Liu Z. (2018). MiR-21 improves invasion and migration of drug-resistant lung adenocarcinoma cancer cell and transformation of EMT through targetingHBP1. Cancer Med..

[26] Li G., Li M., Hu J., Lei R., Xiong H., Ji H., Yin H., Wei Q., Hu G. (2016). The microRNA-182-PDK4 axis regulates lung tumorigenesis by modulating pyruvate dehydrogenase and lipogenesis. Oncogene.

[27] Miko E., Margitai Z., Czimmerer Z., Várkonyi I., Dezso B., Lányi A., Bacsó Z., Scholtz B. (2011). miR-126 inhibits proliferation of small cell lung cancer cells by targeting SLC7A5. FEBS Lett..

[28] Wang Y., Zhao M., Liu J., Sun Z., Ni J., Liu H. (2017). miRNA-125b regulates apoptosis of human non-small cell lung cancer via the PI3K/Akt/GSK3β signaling pathway. Oncol. Rep..

[29] Chen D., Huang J., Zhang K., Pan B., Chen J., De W., Wang R., Chen L. (2014). MicroRNA-451 induces epithelial–mesenchymal transition in docetaxel-resistant lung adenocarcinoma cells by targeting proto-oncogene c-Myc. Eur. J. Cancer.

[30] Raver-Shapira N., Marciano E., Meiri E., Spector Y., Rosenfeld N., Moskovits N., Bentwich Z., Oren M. (2007). Transcriptional Activation of miR-34a Contributes to p53-Mediated Apoptosis. Mol. Cell.

[31] Hermeking H. (2009). The miR-34 family in cancer and apoptosis. Cell Death Differ..

[32] Mizuno S., Bogaard H.J., Gomez-Arroyo J., Alhussaini A., Kraskauskas D., Cool C.D., Voelkel N.F. (2012). MicroRNA-199a-5p Is Associated With Hypoxia-Inducible Factor-1α Expression in Lungs From Patients With COPD. Chest.

[33] Puissegur M.-P., Mazure N.M., Bertero T., Pradelli L.A., Del Grosso S.J., Robbe-Sermesant K., Maurin T., Lebrigand K., Cardinaud B., Hofman V. (2010). miR-210 is overexpressed in late stages of lung cancer and mediates mitochondrial alterations associated with modulation of HIF-1 activity. Cell Death Differ..

[34] Edmonds M.D., Boyd K.L., Moyo T., Mitra R., Duszynski R., Arrate M.P., Chen X., Zhao Z., Blackwell T.S., Andl T. (2015). MicroRNA-31 initiates lung tumorigenesis and promotes mutant KRAS-driven lung cancer. J. Clin. Investig..

[35] Iqbal M.A., Arora S., Prakasam G., Calin G.A., Syed M.A. (2019). MicroRNA in lung cancer: Role, mechanisms, pathways and therapeutic relevance. Mol. Asp. Med..

[36] Hirschey M.D., DeBerardinis R.J., Diehl A.M.E., Drew J.E., Frezza C., Green M.F., Jones L.W., Ko Y.H., Le A., Lea M.A. (2015). Dysregulated metabolism contributes to oncogenesis. Semin. Cancer Biol..

[37] Vanhove K., Derveaux E., Graulus G.-J., Mesotten L., Thomeer M., Noben J.-P., Guedens W., Adriaensens P. (2019). Glutamine Addiction and Therapeutic Strategies in Lung Cancer. Int. J. Mol. Sci..

[38] Chen L.-Q., Hou B.-H., LaLonde S., Takanaga H., Hartung M.L., Qu X.-Q., Guo W.-J., Kim J.-G., Underwood W., Chaudhuri B. (2010). Sugar transporters for intercellular exchange and nutrition of pathogens. Nat. Cell Biol..

[39] Ancey P., Contat C., Meylan E. (2018). Glucose transporters in cancer—From tumor cells to the tumor microenvironment. FEBS J..

[40] Robey R.B., Hay N. (2006). Mitochondrial hexokinases, novel mediators of the antiapoptotic effects of growth factors and Akt. Oncogene.

[41] Mathupala S.P., Rempel A., Pedersen P.L. (2001). Glucose catabolism in cancer cells: Identification and characterization of a marked activation response of the type II hexokinase gene to hypoxic conditions. J. Biol. Chem..

[42] Patra K.C., Wang Q., Bhaskar P.T., Miller L., Wang Z., Wheaton W., Chandel N., Laakso M., Muller W.J., Allen E.L. (2013). Hexokinase 2 Is Required for Tumor Initiation and Maintenance and Its Systemic Deletion Is Therapeutic in Mouse Models of Cancer. Cancer Cell.

[43] Singh A., Happel C., Manna S.K., Acquaah-Mensah G., Carrerero J., Kumar S., Nasipuri P., Krausz K.W., Wakabayashi N., Dewi R. (2013). Transcription factor NRF2 regulates miR-1 and miR-206 to drive tumorigenesis. J. Clin. Investig..

[44] Wong N., Ojo D., Yan J., Tang D. (2015). PKM2 contributes to cancer metabolism. Cancer Lett..

[45] Su Q., Luo S., Tan Q., Deng J., Zhou S., Peng M., Tao T., Yang X. (2019). The role of pyruvate kinase M2 in anticancer therapeutic treatments (Review). Oncol. Lett..

[46] Ji H., Wang J., Guo J., Li Y., Lian S., Guo W., Yang H., Kong F., Zhen L., Guo L. (2016). Progress in the biological function of alpha-enolase. Anim. Nutr..

[47] Hitosugi T., Zhou L., Elf S., Fan J., Kang H.-B., Seo J.H., Shan C., Dai Q., Zhang L., Xie J. (2012). Phosphoglycerate Mutase 1 Coordinates Glycolysis and Biosynthesis to Promote Tumor Growth. Cancer Cell.

[48] Bauer D.E., Hatzivassiliou G., Zhao F., Andreadis C., Thompson C.B. (2005). ATP citrate lyase is an important component of cell growth and transformation. Oncogene.

[49] Hochachka P., Rupert J., Goldenberg L., Gleave M., Kozlowski P. (2002). Going malignant: The hypoxia-cancer connection in the prostate. BioEssays.

[50] Santos C.R., Schulze A. (2012). Lipid metabolism in cancer. FEBS J..

[51] Giró-Perafita A., Sarrats A., Pérez-Bueno F., Oliveras G., Buxó M., Brunet J., Viñas G., Miquel T.P. (2017). Fatty acid synthase expression and its association with clinico-histopathological features in triple-negative breast cancer. Oncotarget.

[52] Wang Y., Zhang X., Tan W., Fu J., Zhang W. (2002). [Significance of fatty acid synthase expression in non-small cell lung cancer]. Zhonghua zhong liu za zhi Chin. J. Oncol..

[53] Agustsson T., Rydén M., Hoffstedt J., Van Harmelen V., Dicker A., Laurencikiene J., Isaksson B., Permert J., Arner P. (2007). Mechanism of Increased Lipolysis in Cancer Cachexia. Cancer Res..

[54] Huang J., Fan X.-X., He J., Pan H., Liyan H., Huang L., Jiang Z., Yao X.-J., Liu L., Leung E.L.-H. (2016). SCD1 is associated with tumor promotion, late stage and poor survival in lung adenocarcinoma. Oncotarget.

[55] Osugi J., Yamaura T., Muto S., Okabe N., Matsumura Y., Hoshino M., Higuchi M., Suzuki H., Gotoh M. (2015). Prognostic impact of the combination of glucose transporter 1 and ATP citrate lyase in node-negative patients with non-small lung cancer. Lung Cancer.

[56] Hanai J.-I., Doro N., Sasaki A.T., Kobayashi S., Cantley L.C., Seth P., Sukhatme V.P. (2011). Inhibition of lung cancer growth: ATP citrate lyase knockdown and statin treatment leads to dual blockade of mitogen-activated protein Kinase (MAPK) and Phosphatidylinositol-3-kinase (PI3K)/AKT pathways. J. Cell. Physiol..

[57] Salvador M.M., de Cedrón M.G., Rubio J.M., Martínez S.F., Martínez R.S., Casado E., de Molina A.R., Sereno M. (2017). Lipid metabolism and lung cancer. Crit. Rev. Oncol..

[58] Feng Y., Xiong Y., Qiao T., Li X., Jia L., Han Y. (2018). Lactate dehydrogenase A: A key player in carcinogenesis and potential target in cancer therapy. Cancer Med..

[59] Xu Z., Liu X., Wang H., Li J., Dai L., Li J., Dong C. (2018). Lung adenocarcinoma cell-derived exosomal miR-21 facilitates osteoclastogenesis. Gene.

[60] Khaidakov M., Mehta J.L. (2012). Oxidized LDL Triggers Pro-Oncogenic Signaling in Human Breast Mammary Epithelial Cells Partly via Stimulation of MiR-21. PLoS ONE.

[61] Zhao J., Zhi Z., Wang C., Xing H., Song G., Yu X., Zhu Y., Wang X., Zhang X., Di Y. (2017). Exogenous lipids promote the growth of breast cancer cells via CD36. Oncol. Rep..

[62] Ni K., Wang D., Xu H., Mei F., Wu C., Liu Z., Zhou B. (2019). miR-21 promotes non-small cell lung cancer cells growth by regulating fatty acid metabolism. Cancer Cell Int..

[63] Wang J., Li Y. (2019). CD36 tango in cancer: Signaling pathways and functions. Theranostics.

[64] Li J.-D., Feng Q.-C., Qi Y., Cui G., Zhao S. (2017). PPARGC1A is upregulated and facilitates lung cancer metastasis. Exp. Cell Res..

[65] Kamata H., Honda S., Maeda S., Chang L., Hirata H., Karin M. (2005). Reactive oxygen species promote TNFalpha-induced death and sustained JNK activation by inhibiting MAP kinase phosphatases. Cell.

[66] Milne J.L.S., Lennarz W.J., Lane M.D. (2013). Structure and Regulation of Pyruvate Dehydrogenases. Encyclopedia of Biological Chemistry.

[67] Yang C., Wang S., Ruan H., Li B., Cheng Z., He J., Zuo Q., Yu C., Wang H., Lv Y. (2019). Downregulation of PDK4 Increases Lipogenesis and Associates with Poor Prognosis in Hepatocellular Carcinoma. J. Cancer.

[68] Vettore L., Westbrook R.L., Tennant D.A. (2020). New aspects of amino acid metabolism in cancer. Br. J. Cancer.

[69] Matés J.M., Segura J.A., Martín-Rufián M., Campos-Sandoval J.A., Alonso F.J., Márquez J. (2013). Glutaminase isoenzymes as key regulators in metabolic and oxidative stress against cancer. Curr. Mol. Med..

[70] Weinberg S.E., Chandel N.S. (2015). Targeting mitochondria metabolism for cancer therapy. Nat. Chem. Biol..

[71] Kanai Y., Segawa H., Miyamoto K.-I., Uchino H., Takeda E., Endou H. (1998). Expression Cloning and Characterization of a Transporter for Large Neutral Amino Acids Activated by the Heavy Chain of 4F2 Antigen (CD98). J. Biol. Chem..

[72] Shimobayashi M., Hall M.N. (2016). Multiple amino acid sensing inputs to mTORC1. Cell Res..

[73] Guo C., Sah J.F., Beard L., Willson J.K.V., Markowitz S.D., Guda K. (2008). The noncoding RNA, miR-126, suppresses the growth of neoplastic cells by targeting phosphatidylinositol 3-kinase signaling and is frequently lost in colon cancers. Genes Chromosom. Cancer.

[74] Koundouros N., Poulogiannis G. (2018). Phosphoinositide 3-Kinase/Akt Signaling and Redox Metabolism in Cancer. Front. Oncol..

[75] Fruman D.A., Chiu H., Hopkins B.D., Bagrodia S., Cantley L.C., Abraham R.T. (2017). The PI3K Pathway in Human Disease. Cell.

[76] Chen C.-Y., Chen J., He L., Stiles B.L. (2018). PTEN: Tumor Suppressor and Metabolic Regulator. Front. Endocrinol..

[77] Manning B.D., Toker A. (2017). AKT/PKB Signaling: Navigating the Network. Cell.

[78] Semenza G.L. (2003). Targeting HIF-1 for cancer therapy. Nat. Rev. Cancer.

[79] Gregory M.A., Qi Y., Hann S.R. (2003). Phosphorylation by Glycogen Synthase Kinase-3 Controls c-Myc Proteolysis and Subnuclear Localization. J. Biol. Chem..

[80] Ben-Sahra I., Hoxhaj G., Ricoult S.J.H., Asara J.M., Manning B.D. (2016). mTORC1 induces purine synthesis through control of the mitochondrial tetrahydrofolate cycle. Science.

[81] Porstmann T., Griffiths B., Chung Y.-L., Delpuech O., Griffiths J.R., Downward J., Schulze A. (2005). PKB/Akt induces transcription of enzymes involved in cholesterol and fatty acid biosynthesis via activation of SREBP. Oncogene.

[82] Liu B., Wu X., Liu B., Wang C., Liu Y., Zhou Q., Xu K. (2012). MiR-26a enhances metastasis potential of lung cancer cells via AKT pathway by targeting PTEN. Biochim. Biophys. Acta (BBA) Mol. Basis Dis..

[83] Dai Q., Li N., Zhou X. (2017). Increased miR-21a provides metabolic advantages through suppression of FBP1 expression in non-small cell lung cancer cells. Am. J. Cancer Res..

[84] Lv X., Yao L., Zhang J., Han P., Li C. (2016). Inhibition of microRNA-155 sensitizes lung cancer cells to irradiation via suppression of HK2-modulated glucose metabolism. Mol. Med. Rep..

[85] Doble B.W., Woodgett J.R. (2003). GSK-3: Tricks of the trade for a multi-tasking kinase. J. Cell Sci..

[86] Peng Y., Zhang X., Feng X., Fan X., Jin Z. (2016). The crosstalk between microRNAs and the Wnt/β-catenin signaling pathway in cancer. Oncotarget.

[87] Kim J.-T., Lee S.Y., Lim S.H., Yu S.-L., Ku G.W., Jeong I.B., Kwon S.J., Kim J.H., Kwon S.J., Kang J. (2018). microRNA 181a-5p Reprogramed Glucose and Lipid Metabolism in Non- Small Cell Lung Cancer. J. Cancer Sci. Clin. Oncol..

[88] Pelengaris S., Khan M., Evan G. (2002). c-MYC: More than just a matter of life and death. Nat. Rev. Cancer.

[89] Miller D.M., Thomas S.D., Islam A., Muench D., Sedoris K. (2012). c-Myc and Cancer Metabolism. Clin. Cancer Res..

[90] Dong Y., Tu R., Liu H., Qing G. (2020). Regulation of cancer cell metabolism: Oncogenic MYC in the driver’s seat. Signal Transduct. Target. Ther..

[91] Liu J., Zhang C., Hu W., Feng Z. (2015). Tumor suppressor p53 and its mutants in cancer metabolism. Cancer Lett..

[92] Schwartzenberg-Bar-Yoseph F., Armoni M., Karnieli E. (2004). The Tumor Suppressor p53 Down-Regulates Glucose Transporters GLUT1 and GLUT4 Gene Expression. Cancer Res..

[93] Contractor T., Harris C.R. (2012). p53 Negatively Regulates Transcription of the Pyruvate Dehydrogenase Kinase Pdk2. Cancer Res..

[94] Kondoh H., Lleonart M.E., Gil J., Wang J., Degan P., Peters G., Martinez D., Carnero A., Beach D. (2005). Glycolytic enzymes can modulate cellular life span. Cancer Res..

[95] Zhang C., Lin M., Wu R., Wang X., Yang B., Levine A.J., Hu W., Feng Z. (2011). Parkin, a p53 target gene, mediates the role of p53 in glucose metabolism and the Warburg effect. Proc. Natl. Acad. Sci. USA.

[96] Liu J., Zhang C., Hu W., Feng Z. (2019). Tumor suppressor p53 and metabolism. J. Mol. Cell Biol..

[97] Chang T.-C., Wentzel E.A., Kent O.A., Ramachandran K., Mullendore M., Lee K.H., Feldmann G., Yamakuchi M., Ferlito M., Lowenstein C.J. (2007). Transactivation of miR-34a by p53 Broadly Influences Gene Expression and Promotes Apoptosis. Mol. Cell.

[98] Salem A., Asselin M.-C., Reymen B., Jackson A., Lambin P., West C.M.L., O’Connor J.P.B., Faivre-Finn C. (2017). Targeting Hypoxia to Improve Non–Small Cell Lung Cancer Outcome. J. Natl. Cancer Inst..

[99] Ren W., Mi D., Yang K., Cao N., Tian J., Li Z., Ma B. (2013). The expression of hypoxia-inducible factor-1α and its clinical significance in lung cancer: A systematic review and meta-analysis. Swiss Med. Wkly..

[100] Muz B., De La Puente P., Azab F., Azab A.K. (2015). The role of hypoxia in cancer progression, angiogenesis, metastasis, and resistance to therapy. Hypoxia.

[101] Chan S.Y., Zhang Y.Y., Hemann C., Mahoney C.E., Zweier J.L., Loscalzo J. (2009). MicroRNA-210 controls mitochondrial metabolism during hypoxia by repressing the iron-sulfur cluster assembly proteins ISCU1/2. Cell Metab..

[102] Denko N.C. (2008). Hypoxia, HIF1 and glucose metabolism in the solid tumour. Nat. Rev. Cancer.

[103] Zheng X., Linke S., Dias J.M., Gradin K., Wallis T.P., Hamilton B.R., Gustafsson M., Ruas J.L., Wilkins S., Bilton R.L. (2008). Interaction with factor inhibiting HIF-1 defines an additional mode of cross-coupling between the Notch and hypoxia signaling pathways. Proc. Natl. Acad. Sci. USA.

[104] Zhang N., Fu Z., Linke S., Chicher J., Gorman J.J., Visk D., Haddad G.G., Poellinger L., Peet D.J., Powell F. (2010). The Asparaginyl Hydroxylase Factor Inhibiting HIF-1α Is an Essential Regulator of Metabolism. Cell Metab..

[105] Zhu B., Cao X., Zhang W., Pan G., Yi Q., Zhong W., Yan D. (2019). MicroRNA-31-5p enhances the Warburg effect via targeting FIH. FASEB J..

[106] Gibbons D.L., Byers L.A., Kurie J.M. (2014). Smoking, p53 Mutation, and Lung Cancer. Mol. Cancer Res..

[107] Jiang S., Wang R., Yan H., Jin L., Dou X., Chen N. (2016). MicroRNA-21 modulates radiation resistance through upregulation of hypoxia-inducible factor-1α-promoted glycolysis in non-small cell lung cancer cells. Mol. Med. Rep..

[108] Byun Y., Choi Y.-C., Jeong Y., Lee G., Yoon S., Jeong Y., Yoon J., Baek K. (2019). MiR-200c downregulates HIF-1α and inhibits migration of lung cancer cells. Cell. Mol. Biol. Lett..

[109] Cha S.-T., Chen P.-S., Johansson G., Chu C.-Y., Wang M.-Y., Jeng Y.-M., Yu S.-L., Chen J.-S., Chang K.-J., Jee S.-H. (2010). MicroRNA-519c Suppresses Hypoxia-Inducible Factor-1α Expression and Tumor Angiogenesis. Cancer Res..

[110] Esquela-Kerscher A., Slack F.J. (2006). Oncomirs—microRNAs with a role in cancer. Nat. Rev. Cancer.

[111] Fortunato O., Boeri M., Verri C., Moro M., Sozzi G. (2014). Therapeutic Use of MicroRNAs in Lung Cancer. BioMed Res. Int..

[112] Van Rooij E., Olson E.N. (2007). MicroRNAs: Powerful new regulators of heart disease and provocative therapeutic targets. J. Clin. Investig..

[113] Ahn Y.-H., Ko Y.H. (2020). Diagnostic and Therapeutic Implications of microRNAs in Non-Small Cell Lung Cancer. Int. J. Mol. Sci..

[114] Finnerty J.R., Wang W.X., Hébert S.S., Wilfred B.R., Mao G., Nelson P.T. (2010). The miR-15/107 group of microRNA genes: Evolutionary biology, cellular functions, and roles in human diseases. J. Mol. Biol..

[115] Wu Y., Crawford M., Mao Y., Lee R.J., Davis I.C., Elton T.S., Lee L.J., Nana-Sinkam S.P. (2013). Therapeutic Delivery of MicroRNA-29b by Cationic Lipoplexes for Lung Cancer. Mol. Ther. Nucleic Acids.

[116] Rai K., Takigawa N., Ito S., Kashihara H., Ichihara E., Yasuda T., Shimizu K., Tanimoto M., Kiura K. (2011). Liposomal Delivery of MicroRNA-7–Expressing Plasmid Overcomes Epidermal Growth Factor Receptor Tyrosine Kinase Inhibitor-Resistance in Lung Cancer Cells. Mol. Cancer Ther..

[117] Wiggins J.F., Ruffino L., Kelnar K., Omotola M., Patrawala L., Brown D., Bader A.G. (2010). Development of a Lung Cancer Therapeutic Based on the Tumor Suppressor MicroRNA-34. Cancer Res..

[118] Cortez M.A., Valdecanas D., Zhang X., Zhan Y., Bhardwaj V., Calin G.A., Komaki R., Giri D.K., Quini C.C., Wolfe T. (2014). Therapeutic Delivery of miR-200c Enhances Radiosensitivity in Lung Cancer. Mol. Ther..

[119] Vázquez-Ríos A.J., Molina-Crespo Á., Bouzo B.L., López-López R., Moreno-Bueno G., De La Fuente M. (2019). Exosome-mimetic nanoplatforms for targeted cancer drug delivery. J. Nanobiotechnol..

[120] Trang P., Wiggins J.F., Daige C.L., Cho C., Omotola M., Brown D., Weidhaas J.B., Bader A.G., Slack F.J. (2011). Systemic Delivery of Tumor Suppressor microRNA Mimics Using a Neutral Lipid Emulsion Inhibits Lung Tumors in Mice. Mol. Ther..

[121] Barger J.F., Nana-Sinkam S.P. (2015). MicroRNA as tools and therapeutics in lung cancer. Respir. Med..

[122] Ling H., Fabbri M., Calin G.A. (2013). MicroRNAs and other non-coding RNAs as targets for anticancer drug development. Nat. Rev. Drug Discov..

[123] Soriano A., Jubierre L., Almazán-Moga A., Molist C., Roma J., de Toledo J.S., Gallego S., Segura M.F. (2013). microRNAs as pharmacological targets in cancer. Pharmacol. Res..

[124] Lujambio A., Ropero S., Ballestar E., Fraga M.F., Cerrato C., Setién F., Casado S., Suarez-Gauthier A., Sanchez-Cespedes M., Gitt A. (2007). Genetic unmasking of an epigenetically silenced microRNA in human cancer cells. Cancer Res..

[125] Krützfeldt J., Rajewsky N., Braich R., Rajeev K.G., Tuschl T., Manoharan M., Stoffel M. (2005). Silencing of microRNAs in vivo with ‘antagomirs’. Nature.

[126] Ørom U.A., Kauppinen S., Lund A.H. (2006). LNA-modified oligonucleotides mediate specific inhibition of microRNA function. Gene.

[127] Gebert L.F.R., Rebhan M.A.E., Crivelli S.E.M., Denzler R., Stoffel M., Hall J. (2014). Miravirsen (SPC3649) can inhibit the biogenesis of miR-122. Nucleic Acids Res..

[128] Janssen H.L.A., Reesink H.W., Lawitz E.J., Zeuzem S., Rodriguez-Torres M., Patel K., Van Der Meer A.J., Patick A.K., Chen A., Zhou Y. (2013). Treatment of HCV Infection by Targeting MicroRNA. N. Engl. J. Med..

[129] Narita M., Shimura E., Nagasawa A., Aiuchi T., Suda Y., Hamada Y., Ikegami D., Iwasawa C., Arakawa K., Igarashi K. (2017). Chronic treatment of non-small-cell lung cancer cells with gefitinib leads to an epigenetic loss of epithelial properties associated with reductions in microRNA-155 and -200c. PLoS ONE.

[130] Li B., Ding C.-M., Li Y.-X., Peng J.-C., Geng N., Qin W.-W. (2018). Over-regulation of microRNA-133b inhibits cell proliferation of cisplatin-induced non-small cell lung cancer cells through PI3K/Akt and JAK2/STAT3 signaling pathway by targeting EGFR. Oncol. Rep..

[131] Zhang Y.-X., Yue Z., Wang P.-Y., Li Y.-J., Xin J.-X., Pang M., Zheng Q.-Y., Xie S.-Y. (2013). Cisplatin upregulates MSH2 expression by reducing miR-21 to inhibit A549 cell growth. Biomed. Pharmacother..

[132] Jin H., Qiao F., Wang Y., Xu Y., Shang Y. (2015). Curcumin inhibits cell proliferation and induces apoptosis of human non-small cell lung cancer cells through the upregulation of miR-192-5p and suppression of PI3K/Akt signaling pathway. Oncol. Rep..

[133] Jiang L.-L., Zhou S.-J., Zhang X.-M., Chen H.-Q., Liu W. (2016). Sulforaphane suppresses in vitro and in vivo lung tumorigenesis through downregulation of HDAC activity. Biomed. Pharmacother..

[134] Yagishita S., Fujita Y., Kitazono S., Ko R., Nakadate Y., Sawada T., Kitamura Y., Shimoyama T., Maeda Y., Takahashi F. (2015). Chemotherapy-Regulated microRNA-125–HER2 Pathway as a Novel Therapeutic Target for Trastuzumab-Mediated Cellular Cytotoxicity in Small Cell Lung Cancer. Mol. Cancer Ther..

[135] Zhang S., Zhang C., Li Y., Wang P., Yue Z., Xie S. (2011). miR-98 regulates cisplatin-induced A549 cell death by inhibiting TP53 pathway. Biomed. Pharmacother..

[136] Xu R., Shen H., Guo R., Sun J., Gao W., Shu Y. (2012). Combine therapy of gefitinib and fulvestrant enhances antitumor effects on NSCLC cell lines with acquired resistance to gefitinib. Biomed. Pharmacother..

[137] Zhou D.-H., Wang X., Feng Q. (2014). EGCG enhances the efficacy of cisplatin by downregulating HSA-miR-98-5p in NSCLC A549 cells. Nutr. Cancer..

